# Antidepressants in Bipolar Depression: From Neurotransmitter Mechanisms to Clinical Challenges

**DOI:** 10.62641/aep.v53i3.1880

**Published:** 2025-05-05

**Authors:** Simone Pardossi, Andrea Fagiolini, Alessandro Cuomo

**Affiliations:** ^1^Department of Molecular Medicine, University of Siena School of Medicine, 53100 Siena, Italy

**Keywords:** antidepressants, bipolar disorder, bipolar depression, treatment, psychopharmacology

## Abstract

Bipolar disorder (BD) is characterized by the occurrence of manic/hypomanic and depressive episodes, with the latter having a significant impact on morbidity, mortality and overall quality of life. Current guidelines for bipolar depression provide limited treatment options, with only a few approved therapies. Despite these limitations, approximately 50–60% of individuals diagnosed with BD are prescribed antidepressants. However, the use of these medications remains controversial due to risks of manic induction, rapid cycling, and symptom destabilization. This review explores the neurotransmitter mechanisms underpinning the phases of BD, focusing on monoamines and assessing the efficacy and safety of different antidepressant medications in the treatment of bipolar depression. Norepinephrine and dopamine have been identified as neurotransmitters associated with both depressive and manic poles, with a proposed deficit in depression and an increase in mania. The evidence indicates that serotonin is deficient during depressive phases, yet its imbalance also manifests in mania. Selective serotonin reuptake inhibitors (SSRIs), which primarily increase serotonin levels, are generally safer than tricyclic antidepressants (TCAs) and show promising—though not definitive—results, especially when combined with mood stabilizers. Other newer-generation antidepressants may also have potential for the treatment of bipolar depression. The heterogeneity of mood disorders poses a significant challenge in the diagnosis of BD, which is often ambiguous and complex. The natural mood fluctuations associated with BD, in conjunction with the frequent comorbidities such as anxiety, render the treatment of this condition particularly challenging, particularly in the context of antidepressant therapy. While clinical trials are conducted with the utmost rigor, they frequently fail to account for the intricacies of the real-world context due to the strict inclusion criteria. The identification of predictors of effective antidepressant use, such as symptom severity and comorbid conditions, has the potential to enhance treatment outcomes. Future research should aim to identify individualized predictors and deepen understanding of mood disorder spectra to optimize antidepressant use in bipolar depression.

## Introduction

### Bipolar Disorder and Bipolar Depression

Bipolar disorder (BD) is a well-established psychiatric condition, as defined by the Diagnostic and Statistical Manual of Mental Disorders, Fifth Edition, Text Revision (DSM-5-TR). The condition is characterized by the occurrence of alternating manic/hypomanic and depressive episodes, interspersed with periods of remission [1, 2]. This intermittent nature of the condition poses significant challenges to pharmacological treatment [2, 3]. The management of BD involves the treatment of manic and hypomanic episodes, the treatment of depressive phases, and the implementation of prophylaxis to prevent relapses in either direction [2, 3].

Each episode exhibits diverse characteristics, varying both between patients and within the same patient across episodes [1, 2]. Depressive episodes in BD involve cognitive, affective, and neurovegetative symptoms, sometimes accompanied by changes in thought form and content [1]. Conversely, manic and hypomanic episodes typically manifest as mood alterations, disrupted sleep, increased energy, and, in severe cases, the onset of psychotic symptoms [4, 5]. In light of a dimensional approach to BD, treatment is frequently oriented towards targeting disparate symptoms, and on occasion, even contrasting ones, such as mixed symptoms [6, 7, 8]. BD frequently co-occurs with other disorders, including personality disorders, substance abuse, and anxiety disorders [9, 10].

Bipolar depression is of particular concern due to its association with significant morbidity and mortality. This is due to the fact that depressive episodes are far more frequent and typically more prolonged than manic episodes [5, 11]. Individuals with BD spend up to three times more time in depressive states than in manic or hypomanic episodes [5, 11]. The consequences of bipolar depression are far-reaching, resulting in elevated levels of disability and diminished quality of life when compared to the manic phases of BD [12]. This is evidenced by the adverse effects on cognitive function, psychosocial outcomes, and employment status, frequently leading to socioeconomic disadvantages [13, 14]. Depressive symptoms in BD, including anhedonia, psychomotor retardation, and pervasive mood dysregulation, contribute to high rates of relapse and hospitalization, significantly impacting the individual’s social and occupational functioning over time [13]. Notably, suicidal ideation and behaviors are markedly elevated during depressive episodes, with rates approximately 20–30 times higher than in the general population [14], contributing to the elevated mortality risk in BD [14]. The significance of depressive episodes in BD is widely recognized, not only due to their comparatively longer duration over the course of patients’ lives but also due to their significant impact on functioning [12].

### Bipolar Depression Treatment–Current Status

Regarding the treatment of BD, long-term management involves the use of mood stabilizers such as lithium, valproate, and lamotrigine, as well as atypical antipsychotics, including quetiapine, aripiprazole, lurasidone, and cariprazine [2].

Nevertheless, the treatment of bipolar depression remains a significant challenge, with a limited number of approved medications specifically approved for this phase. In countries such as the United States, several medications have been approved for the treatment of bipolar depression in adults, including quetiapine [15], lurasidone [16], the olanzapine-fluoxetine combination [17], cariprazine [18], and lumateperone [19].

Conversely, the European Medicines Agency (EMA) has approved only quetiapine for bipolar depression [20]. This discrepancy engenders challenges for clinicians, who often lack clear and locally applicable guidelines for effectively managing this debilitating condition. Consequently, it has been observed that approximately 50–60% of patients with BD are prescribed antidepressants, with notable variation across healthcare systems [21]. For instance, a Danish study found that approximately 50% of bipolar patients continued using antidepressants for at least two years after diagnosis, with a significant proportion receiving antidepressants without concurrent mood stabilizers [22], raising clinical concerns about the risk of manic induction [23]. A similar trend was observed in the United States, where antidepressants were prescribed in 57.5% of outpatient bipolar visits by 2016, reflecting a two-decade trend toward increased use, often without mood-stabilizing agents [24]. The utilization of antidepressants in bipolar depression remains a subject of considerable controversy. While antidepressants are widely utilized for the treatment of major depressive disorder, their efficacy in bipolar depression remains a subject of debate. This review aims to explore the monoaminergic underpinnings of BD phases and analyze existing evidence regarding the use of antidepressants from various classes in the treatment of bipolar depression.

## Monoamines in Bipolar Disorder

The neurobiology of BD is characterized by a fundamental aspect of neurotransmitter dysregulation. Within the framework of the monoaminergic theory of mood disorders, extensive research has been conducted on the role of dopamine, norepinephrine, and serotonin in affective disorders [25]. Elevated urinary levels of norepinephrine and dopamine have been linked to the onset of mania and the transition into manic episodes [26].

The role of dopamine in BD has been a cornerstone of its neurobiological understanding, particularly in relation to the contrasting states of mania and depression. Manic episodes are strongly associated with hyperdopaminergic activity, characterized by elevated dopamine synthesis and transmission within the mesolimbic and mesocortical pathways, key regions involved in reward processing and cognitive control [27]. This hyperactivity is associated with increased reward sensitivity, impulsivity, and risk-taking behaviors frequently observed during manic phases [27, 28]. Neuroimaging studies have demonstrated increased dopamine receptor availability, particularly at D2/3 receptors, in individuals experiencing mania [27]. These findings are further supported by the efficacy of antidopaminergic agents, which directly target these pathways to reduce manic symptoms [27, 29, 30]. Animal models confirm these observations. The pharmacological induction of hyperdopaminergic states, such as through amphetamine administration or dopamine transporter knockout, leads to manic-like behaviors, including hyperlocomotion and increased exploratory activity [27, 31]. Conversely, lesions in dopaminergic regions, such as the ventral tegmental area (VTA) or the substantia nigra, result in depressive-like behaviors, thereby reinforcing the bidirectional role of dopamine dysregulation in BD [27]. Studies in humans further corroborate dopamine’s role. A study using amphetamines has provided direct evidence of this link [32]. For instance, research employing positron emission tomography (PET) imaging has shown that dopamine release in the anteroventral striatum (AVS)—a region comprising the nucleus accumbens, ventromedial caudate, and anteroventral putamen—correlates strongly with the euphoric response induced by amphetamines [32]. This relationship highlights the AVS as a critical region for dopamine’s role in hedonic and emotional processing. Notably, this correlation was not observed in other regions, such as the dorsal caudate, underscoring the unique sensitivity of the ventral striatum to dopamine release and its association with manic-like symptoms [32]. Furthermore, lithium, a prominent therapeutic option for BD, is believed to stabilize mood, at least in part, by facilitating the metabolic conversion of dopamine to its inactive form [33]. This reduces dopaminergic activity and helps to regulate manic symptoms [32, 33].

In contrast, depressive episodes have been linked to hypodopaminergic states, marked by reduced dopamine synthesis, release, and receptor activity in key regions implicated with reward and motivation, such as the striatum and prefrontal cortex [27, 29]. Elevated dopamine transporter (DAT) levels, observed in neuroimaging studies of individuals with bipolar depression, may contribute to the diminished dopaminergic tone observed in these patients [27]. This hypodopaminergic state aligns with hallmark symptoms of bipolar depression, including anhedonia, low energy, and reduced motivation [27, 29].

The dopamine hypothesis of bipolar disorder has evolved into a “failure of homeostasis” model, proposing that intrinsic dysregulation of dopamine receptor and transporter mechanisms contributes to the pathophysiology of bipolar disorder [27, 29].

Elevated norepinephrine levels have been observed during manic episodes, contributing to the physiological hyperarousal and increased goal-directed activity characteristic of mania [28]. The metabolite of norepinephrine, 3-methoxy-4-hydroxyphenylglycol (MHPG), is also consistently elevated in individuals experiencing manic states, suggesting a strong correlation between noradrenergic hyperactivity and manic symptoms [26, 34]. Studies further indicate that patients in a manic episode exhibit higher levels of MHPG than those in depressive or remission states [26, 35, 36]. This finding lends further support to the hypothesis that norepinephrine plays a central role in manic episodes [26, 35, 36]. During depressive phases, reduced norepinephrine activity, particularly in the prefrontal cortex and limbic system—regions critical for mood and emotional processing—is linked to depressive symptoms. These regions are dependent on adequate noradrenergic signaling for optimal functionality [37].

Serotonin also plays a key role in the pathophysiology of BD, particularly during depressive episodes. A reduction in central serotonergic activity is a typical feature of bipolar depression, and is associated with a range of symptoms, including low mood, anhedonia and decreased energy [28, 38, 39]. The findings of numerous cerebrospinal fluid (CSF) studies indicate that individuals presenting with depressive symptoms exhibit consistently reduced levels of serotonin and its metabolite, 5-Hydroxyindoleacetic acid (5-HIAA) [28, 38]. This evidence underscores the importance of serotonin deficiency in the pathogenesis of depression. Post-mortem studies have also demonstrated reduced availability of serotonin transporter (SERT) in the brainstem of individuals with depressive disorders, suggesting that impaired serotonergic transmission may serve as a trait marker predisposing individuals to mood dysregulation [40]. The role of serotonin in mania, however, is not fully elucidated. Some studies report increased serotonin turnover, while others suggest diminished serotonergic responsiveness during manic phases [38]. This inconsistency suggests the possibility of a modulatory effect, whereby serotonin exerts an indirect influence on the severity of manic symptoms through interactions with other neurotransmitters, particularly dopamine [38, 39]. Given that serotonin plays a role in regulating impulsivity and response to external stimuli, it can be postulated that dysregulation of this neurotransmitter could impair behavioral control, thereby exacerbating both manic and depressive symptoms when serotonin levels are not properly balanced [39, 41, 42]. Despite these findings, recent research on serotonin’s role in BD remains limited, especially regarding its direct effects on mood cycling.

## Efficacy of Various Antidepressants in Bipolar Depression

The efficacy of antidepressants in treating bipolar depression has been investigated across different pharmacological classes, including selective serotonin reuptake inhibitors (SSRIs), serotonin-norepinephrine reuptake inhibitors (SNRIs), tricyclic antidepressants (TCAs), other antidepressants, and novel agents such as esketamine. Nevertheless, the effectiveness of these medications remains a subject of debate. This is due to the heterogeneous responses of patients with BD and the potential risks of manic induction, rapid cycling, and symptom destabilization.

In comparison to the extant literature on SSRIs and SNRIs, there is a paucity of recent studies on the use of tricyclic antidepressants in BD. This paucity of research can be attributed, at least in part, to the recognized high risk of inducing mood switches with TCAs [43]. Indeed, a 2009 study demonstrated that patients with BD who were treated with TCAs exhibited a significantly higher risk of experiencing a switch in their mood state compared to those who were treated with other antidepressant medications [44].

### Selective Serotonin Reuptake Inhibitors (SSRIs) and Serotonin-Norepinephrine Reuptake Inhibitors (SNRIs) in Monotherapy

SSRIs are among the most commonly prescribed antidepressant medications. Generally, SSRIs inhibit the SERT, thereby increasing serotonin levels in the synaptic cleft [45]. They may also impact neuroplasticity and neuroinflammation, though these effects remain secondary to their primary serotonergic mechanism [46, 47]. However, in consideration of the monoaminergic theory of depression, their primary effect is to increase serotonin levels [44]. At medium to high doses, some SSRIs might influence other neurotransmitters. For instance, sertraline has been shown to modulate dopamine balance [39, 48], while fluoxetine may influence the norepinephrine transporter (NET) [49]. However, at minimal therapeutic doses, most SSRIs primarily act on the SERT, resulting in increased serotonin levels [46, 47].

SNRIs similarly increase serotonin levels but also target norepinephrine by blocking the NET, particularly at higher doses [50]. For instance, venlafaxine initially acts predominantly as a SERT inhibitor and only inhibits NET at increased dosages [51].

A paucity of randomized trials exists that assess the efficacy of SSRIs and SNRIs in the treatment of bipolar depression (Table 1, Ref. [52, 53, 54, 55, 56, 57]). A notable early study compared bipolar patients treated with fluoxetine (10–30 mg/day), olanzapine (5–20 mg/day), the fluoxetine/olanzapine combination (10–40 mg/day/5–15 mg/day), or placebo. A significant reduction in depressive symptoms was observed in all groups, with no evidence of increased manic symptoms. However, the primary limitation of this study was its relatively small sample size [52].

**Table 1.** **Randomized controlled trials on monotherapy with selective serotonin reuptake inhibitors (SSRIs) or serotonin-norepinephrine reuptake inhibitors (SNRIs) for the treatment of bipolar depression**.

Treatment		Design	Antidepressant efficacy	Effect of the antidepressant on manic symptoms
SSRIs				
	Fluoxetine	Comparison of fluoxetine with olanzapine and the combination fluoxetine/olanzapine	Significant reduction in depressive symptoms in all three groups	No significant increase in manic symptoms
	10–30 mg/day			
	[52]			
	Escitalopram	Comparison of escitalopram with placebo	Significant reduction in depressive symptoms in patients treated with escitalopram	No significant increase in manic symptoms
	10 mg/day			
	[53]			
	Paroxetine	Comparison of paroxetine with quetiapine and with placebo	No significant reduction in depressive symptoms in the paroxetine group compared to the placebo group	No significant increase in manic symptoms
	20 mg/day			
	[54]			
	Sertraline	Comparison of sertraline with lithium and the lithium/sertraline combination	No significant differences in antidepressant efficacy were identified across the three groups	No significant increase in manic symptoms
	100 mg/day			
	[57]			
SNRIs				
	Venlafaxine	Comparison of venlafaxine with lithium	Venlafaxine was more effective than lithium in treating depressive symptoms	No significant increase in manic symptoms
	37.5 mg/day, increased by 37.5–75 mg per week			
	Amsterdam and Shults J [55]			
	Venlafaxine 75 mg/day	Comparison of venlafaxine with bupropion	Venlafaxine was as effective as bupropion in treating depressive symptoms	No significant increase in manic symptoms
	[56]			

Another study evaluated escitalopram (10 mg/day) in comparison to placebo. At the three-month mark, patients treated with escitalopram exhibited a reduction in depressive symptoms and an improvement in functioning, accompanied by a decline in the number of days with elevated mood [53]. However, the relatively small sample size represents a limitation.

In a subsequent study, the efficacy of monotherapy with paroxetine (20 mg/day) was compared to that of quetiapine (300–600 mg/day). Following an eight-week period, paroxetine did not significantly improve depressive symptoms relative to placebo. However, paroxetine demonstrated superior efficacy in reducing anxiety symptoms, with no significant manic switches reported [54].

A further study compared bipolar patients treated with lithium monotherapy to those treated with venlafaxine monotherapy. The study revealed that venlafaxine was more effective in treating depressive symptoms, while there were no statistically significant differences in hypomanic symptoms between the two groups. It is noteworthy that venlafaxine was initiated at a dosage of 37.5 mg/day and subsequently increased in a gradual manner, with increments of 37.5–75 mg per week [55]. Another recent study demonstrated the safety and efficacy of venlafaxine at 75 mg/day, showing no increased risk of manic episodes [56].

Sertraline has also been considered as a treatment option. A recent study compared bipolar patients with depressive symptoms across three groups: sertraline monotherapy, lithium monotherapy, and a combination of lithium and sertraline (in sertraline monotherapy, most patients received 100 mg/day) [57]. No patients experienced a manic switch, although some exhibited hypomanic symptoms, with no statistically significant differences among the groups. Similarly, no significant differences in antidepressant efficacy were identified across the three groups [57].

### Antidepressants in Combination With Antipsychotics/Mood Stabilizers

In clinical practice, antidepressants are frequently prescribed in conjunction with other medications, particularly for patients with BD. The utilization of mood stabilizers is an essential consideration in such cases. The findings from meta-analyses remain somewhat inconsistent. A meta-analysis conducted in 2016 [58] indicated that patients treated with an antidepressant (in combination with a mood stabilizer and antipsychotic) showed a slight yet statistically significant improvement in depression severity scores compared to those receiving placebo (alongside a mood stabilizer). However, no significant differences were observed in response or remission rates. The risk of switching to mania or hypomania was not elevated in the short term; it became significant only over longer periods (52 weeks), suggesting that antidepressants should be used cautiously and for short durations.

A 2022 meta-analysis [59] encompassed randomized controlled trials (RCTs) on adult patients experiencing a depressive phase within BD. The analysis compared patients who were prescribed mood stabilizers or antipsychotics exclusively with those also taking antidepressants, including fluoxetine, sertraline, citalopram, paroxetine, and others. While some trials reported superior outcomes in the antidepressant group, the meta-analysis did not reveal statistically significant overall improvement. However, a subgroup analysis found that trials incorporating antipsychotics (instead of mood stabilizers) demonstrated better outcomes in the antidepressant group [59]. Importantly, the analysis did not indicate an increased risk of switching to mania in the antidepressant group. Nevertheless, the duration of the studies (6 to 26 weeks) precludes conclusions regarding long-term safety and efficacy. In 2023, a study investigating the efficacy of bupropion or escitalopram for maintenance therapy following a depressive episode in bipolar patients found no long-term benefits. On the contrary, there was a higher incidence of hypomanic and manic symptoms with continued antidepressant therapy [60].

More recently, a 2024 meta-analysis [61] demonstrated an increased response rate in patients treated with antidepressants (in addition to mood stabilizers or antipsychotics) compared to those treated only with mood stabilizers or antipsychotics. These findings are more consistent with the results of the 2016 meta-analysis [58], but the long-term effects of antidepressants in this population remain inconclusive.

### Potential Role of Other Antidepressants

The treatment of BD has also been explored with antidepressant medications beyond the selective SSRI and SNRI classes. For example, some case reports suggest that trazodone, mirtazapine, and agomelatine may be effective in treating insomnia and may also serve as antidepressants in BD, at low doses and/or in combination with a mood stabilizer [62]. In addition, trazodone has shown potential efficacy in the treatment of psychomotor agitation in patients with BD, especially in its parenteral formulation [63]. A preliminary open-label study indicated that agomelatine was effective in improving sleep and depressive symptoms in bipolar patients. However, hypomanic/manic switching occurred in 14% of patients [64]. Vortioxetine has also shown efficacy in alleviating depressive symptoms associated with BD, although a phase switch rate of 11% was observed in the study sample [65].

Esketamine represents a promising alternative. Unlike traditional monoaminergic antidepressants, esketamine acts on the glutamatergic system, providing a rapid onset of action and a novel mechanism to address depressive symptoms [66]. Although more research is needed to clarify its precise role in bipolar depression, preliminary results suggest that it may be both effective and safe, particularly with regard to minimizing the risk of hypomanic or manic switches [67]. Recent findings from a multicentric naturalistic study have demonstrated the efficacy and safety in patients with bipolar treatment-resistant depression (B-TRD) [68]. In this study, esketamine nasal spray significantly reduced depressive symptoms, with improvements observed as early as the first month of treatment and sustained over three months. Notably, these effects were achieved with a low incidence of manic or hypomanic switches [68]. Furthermore, a recent study involving 45 patients with bipolar depression treated with either intravenous ketamine or intranasal esketamine reported that no patients exhibited hypomanic or manic symptoms during the acute phase of treatment. These findings reinforce the safety profile of esketamine and underscore its potential as an innovative and effective therapeutic option for managing bipolar depression [69].

## Discussion

The topic of treating bipolar depression and maintaining its remission with antidepressants has been extensively examined in the scientific literature, as evidenced by numerous systematic reviews and meta-analyses [43, 59, 70, 71, 72, 73]. This necessity arises from two factors: the relatively high prevalence of BD among psychiatric disorders and the predominance of depressive phases within the disorder that are often debilitating in nature [5, 11]. This pressing need has driven investigations into novel treatments or alternative pharmacological classes, such as antipsychotics, for the management of BD [2]. Consequently, many patients diagnosed with BD are treated with antidepressants, despite limited evidence supporting this approach [73]. However, several considerations must be taken into account when evaluating this discrepancy.

(1) The first challenging step is diagnosis. Bipolar I disorder, bipolar II disorder, and major depression should not be viewed as distinct and separate entities but rather as parts of a mood disorder spectrum [74]. Indeed distinguishing between BD and major depression at the initial evaluation of a depressive episode is often challenging [75, 76]. This diagnostic overlap also explains why this study did not focus exclusively on BD type I or type II. While evidence suggests a higher risk of antidepressant-induced switching in BD type I compared to type II [73], a longitudinal approach to monitoring affective symptom fluctuations may prove more clinically useful [75, 76].

(2) BD is characterized by frequent mood fluctuations and the occurrence of various affective phases, including mixed states [1, 13]. This complicates the evaluation of switches to hypomania or mania in studies involving antidepressants, as it is difficult to determine whether the switch reflects a natural phase shift, antidepressant treatment, or a combination of these factors.

(3) RCTs often fail to capture the complexity and heterogeneity of real-world bipolar disorder presentations [77]. BD often co-occurs with other psychiatric disorders, such as anxiety disorders, which may respond favorably to antidepressants like SSRIs [78]. It is imperative to consider the interplay between the current mood disorder and any psychiatric or medical comorbidities in order to make more informed pharmacological treatment decisions. However, many RCTs exclude patients with comorbidities. For example, studies like Parker’s only included patients who had never been treated with antidepressants, antipsychotics, or mood stabilizers [53]—a rare scenario in clinical practice, where polypharmacy is common. Additionally, some trials have very small sample sizes, such as 10 patients [53], 34 patients [52], and 40 patients [56], which limits their generalizability.

One major concern regarding the use of antidepressants in BD is the risk of mood swings or, more generally, the emergence of hypomanic or manic symptoms [73]. However, this risk has been shown to be lower with SSRIs than with tricyclic antidepressants [72, 73]. To contextualize this, it is essential to review the role of serotonin in affective disorders. SSRIs, particularly at low doses, primarily increase serotonin levels [46]. Animal studies have shown that serotonin depletion leads to increased aggression, maladaptive despair, decreased anxiety, and decreased ability to adapt to new environments [79, 80]. For instance, in an animal model where serotonin depletion induced a manic-like state, treatment with valproate reduced these behavioral changes [80]. Previous research has shown that low serotonin levels significantly correlate with increased aggression typical of antisocial behavior, which is also transdiagnostic in other disorders, such as some cases of manic phases [81, 82].

Studies suggest that serotonin not only modulates aggression but also impulsivity by regulating inhibitory control systems [83]. While these data are insufficient to establish a direct link between serotonin and mood changes, a 2000 review highlighted that cerebrospinal fluid studies, postmortem studies, platelet studies, neuroendocrine challenge tests, and tryptophan depletion studies support the hypothesis that serotonin deficits are involved in mania and that increased serotonergic neurotransmission may exert a mood-stabilizing effect [84]. Mood stabilizers may, in part, act through serotonergic mechanisms [84].

This explanation is undoubtedly simplistic, as SSRIs also have other mechanisms of action that are incompletely understood [46, 47]. It is also important to emphasize that other neurotransmitters, such as dopamine, norepinephrine, glutamate, and gamma-aminobutyric acid (GABA), play important roles in mood phases and mood transitions [25, 85, 86].

While these mechanisms provide a foundation for understanding the role of antidepressants, it remains imperative to offer a variety of treatment options for severe depressive episodes in BD. Although there are no antidepressants specifically indicated for bipolar depression (with the exception of fluoxetine in combination with olanzapine in the United States [17]), the 2018 guidelines for Bipolar I Disorder (BD-I) from the Canadian Network for Mood and Anxiety Treatments (CANMAT) and the International Society for BDs (ISBD) recommend adjunctive treatment with an SSRI or bupropion as a second-line option, while olanzapine-fluoxetine (OFC) is also recommended as a second-line treatment. SNRIs and monoamine oxidase inhibitors (MAOIs) are considered adjunctive third-line treatments, while antidepressant monotherapy is not recommended [87].

Importantly, antidepressants, when used alongside mood stabilizers, are relatively safe in terms of switching risk. Additionally, mood stabilizers like lithium have shown efficacy in treating major depression and preventing suicide, emphasizing their utility even in cases where BD is suspected but not explicitly diagnosed [88].

It is also important to underscore a major limitation in the current body of literature. As noted in the studies cited, RCTs evaluating the use of SSRIs and SNRIs in the treatment of bipolar depression focus predominantly on short-term outcomes, with study durations rarely exceeding a few months [52, 53, 54, 55, 56, 57, 58, 59]. When considering major depressive disorder, many guidelines recommend maintaining antidepressant therapy for at least six months [89]. For example, the American Psychiatric Association recommends continuation therapy for 4–9 months [90], while the Canadian Network for Mood and Anxiety Treatments advises a duration of 6–9 months [91]. In the case of bipolar depression, however, there are no clear guidelines on the optimal duration of antidepressant therapy. A recent study found no significant benefit in continuing treatment with escitalopram or bupropion for 52 weeks compared with an 8-week course [60]. Given the widespread prescription of antidepressants to bipolar patients [21], there is an urgent need for longitudinal studies to evaluate the long-term efficacy and safety of different SSRIs and SNRIs. Such research would provide essential evidence to guide clinicians, particularly in determining how and when to safely discontinue antidepressant therapy in bipolar patients.

As previously stated, it is of critical importance to assess each patient with BD [92]. Establishing universally applicable guidelines remains inherently challenging due to the significant heterogeneity of this disorder and the potential influence of additional variables, such as comorbidities, on treatment plans [92]. For example, even while acknowledging the distribution of mood disorders along a spectrum, it is crucial—particularly for therapeutic decision making—to investigate factors that may help distinguish major depressive disorder from BD [74]. In this regard, Tomasik *et al*. [93] highlighted the potential of biomarker profiling, identifying a metabolomic signature that may differentiate bipolar from unipolar depression during depressive episodes. Moreover, McIntyre *et al*. [94] emphasized the importance of multidimensional clinical characterization in guiding treatment choices. For example, the presence of mixed features, such as co-occurring manic and depressive symptoms, is associated with a poorer response to conventional antidepressants and a higher risk of mood destabilization, necessitating alternative pharmacological strategies such as mood stabilizers or atypical antipsychotics [94]. Neurocognitive impairments, often associated with chronicity or a high frequency of episodes, may impede functional recovery, necessitating interventions aimed at cognitive remediation. Similarly, physical comorbidities, such as metabolic syndrome, and psychiatric comorbidities, such as anxiety disorders, further complicate treatment outcomes and often require integrated approaches that address both mood symptoms and systemic health [94]. The identification of reliable predictors for antidepressant use would undoubtedly be a valuable contribution to the field. In this regard, the study by Fagiolini *et al*. [95] is illustrative, as it identified certain factors in a population of bipolar patients that predicted a positive response with mood stabilizer monotherapy. These factors included being married, having experienced a recent depressive episode, achieving stabilization in a shorter time, and having a higher Young Mania Rating Scale (YMRS) score at baseline. Patients requiring an antidepressant for enhanced remission exhibited higher Hamilton Rating Scale for Depression (HRSD) scores during the initial three weeks treatment period.

Other studies have identified key risk factors for antidepressant-induced switching, including a diagnosis of bipolar I disorder (rather than bipolar II), the presence of mixed features, rapid cycling, the use of tricyclics, and a history of substance abuse, particularly stimulants [73]. Future studies should therefore focus on better characterizing individual patients within the mood disorder spectrum to identify features that might predict the efficacy and safety of antidepressant use.

## Conclusion

The treatment of bipolar depression remains a significant clinical challenge due to the disorder’s complexity and its presentation across a heterogeneous and variable spectrum of symptoms. The lack of clear and effective clinical guidelines further complicates its management. In clinical practice, antidepressants such as SSRIs and SNRIs are commonly used. While some studies suggest potential benefits, the limitations of the current literature—including small sample sizes, short study durations, and the exclusion of comorbidities—highlight the need for cautious interpretation of these findings. Future research should focus on identifying biomarkers to enhance diagnostic precision and predictive factors to guide the decision-making process regarding the initiation of antidepressant therapy and the choice of specific antidepressants. Addressing these gaps would require long-term studies to establish the safety and durability of antidepressant treatments. Ultimately, comprehensive and individualized treatment plans remain essential, integrating pharmacological, psychological, and social interventions to optimize outcomes for patients with bipolar depression.

## Availability of Data and Materials

Not applicable.
